# Haploidentical hematopoietic stem cell transplantation with post-transplant cyclophosphamide in the public Chilean national health system: A single center study

**DOI:** 10.1016/j.htct.2025.103982

**Published:** 2025-09-17

**Authors:** Barbara Puga, Francisca Bass, Javiera Molina, Rafael Benavente, Alejandro Andrade, Alejandro Majlis, María Elena Cabrera, Mi Kwon

**Affiliations:** a Intensive Hematology Unit, Hospital del Salvador, Santiago, Chile; b Internal Medicine Department, University of Chile, Santiago, Chile; c Stem Cell Transplant Unit, Hospital Gregorio Marañón, Madrid, Spain

**Keywords:** Haploidentical transplantation, Peripheral blood stem cell transplantation, Acute graft-versus-host disease, Chronic graft-versus-host disease

## Abstract

**Introduction:**

Haploidentical peripheral stem cell transplantation with post-transplant cyclophosphamide is the most common modality in low-and-middle-income countries. This article reports the consecutive adult patients who received this modality of transplant in a single center in Chile between 2016-2021.

**Methods:**

The primary outcome was overall survival. Secondary outcomes were event-free survival, II-IV acute graft-versus-host disease at Day +100, chronic graft-versus-host disease at two years and cumulative incidence of relapse.

**Results:**

The median age was 25 years (Range: 15-51), and 65 % of patients were male. Ninety-four percent had a neoplastic disease (77/82), with the most common diagnosis being acute lymphoblastic leukemia (57 %). Forty-seven percent proceeded to transplant in the first complete response. Conditioning was mostly myeloablative (96 %). Primary graft failure and poor graft function were observed in 1.2 % and 13 %, respectively with five patients (6.1 %) dying before engraftment. Grade II-III acute graft-versus-host disease was seen in 29 % and chronic graft-versus-host disease was 41 % of the patients. With a median follow-up of 33 months (Range: 1-84), the estimated three-year overall survival and event-free survival were 68.3 % (95 % CI: 59–79 %) and 64.6 % (95 % CI: 55–76 %), respectively. The three-year cumulative incidence of relapse was 23 % (95 % CI: 15–33 %).

**Conclusion:**

These results demonstrate encouraging survival outcomes and acceptable rates of graft-versus-host disease following haploidentical peripheral stem cell transplantation with post-transplant cyclophosphamide, suggesting its potential as a feasible option in low-resource settings.

## Introduction

Allogeneic hematopoietic stem cell transplantation (allo-HSCT) offers a therapeutic option for varied neoplastic and non-neoplastic hematologic diseases. In patients without a matched sibling donor (MSD), alternative sources like HLA-matched unrelated donors (MUD) or umbilical cord, have extended the access to this therapy, but usually associated with a longer latency, and higher complexity and cost. In this context, the option of haploidentical (Haplo) related donors identifies suitable donors for almost 95 % of patients. This alternative source has become a valid option for ethnic minorities and for low- and middle-income countries (LMIC) with limited access to MUD.

Haplo-HSCT was introduced in Chile for the first time at the Luis Calvo Mackenna Children Hospital, with technological support of the St. Jude Children’s Research Hospital providing encouraging results. However, the complexity and cost of ex-vivo T-lymphocyte depletion prevented its widespread use [1,2]. In 2001, investigators from Johns Hopkins Hospital published a Phase I/II trial of Haplo-HSCT with in-vivo T-lymphocyte depletion with post-transplant cyclophosphamide (Haplo-PTCy) [3]. Using this platform, meta-analysis and non-randomized studies have shown similar or even, better results, when Haplo donors are compared with MSD and MUD [4, 5, 6, 7]. These results explain why this modality of hematopoietic stem cell transplantation (HSCT) has increased worldwide [8,9]. In 2020, Sarmiento et al. reported the first local series including 49 cases in a private institution with a three-year overall survival (OS) of 48 % [10]. At the public level, a national adult HSCT program for public health insurance patients, was implemented in 2010. Limited transplantation teams and budgets forced to impose important access limitations in age and donor type. At the beginning only allo-HSCT recipients up to 40 years old with an MSD were included. MUD transplantation was not considered given its high cost and the low chance of successful searches for ethnic minorities, like those of Chile. In 2016, Haplo-PTCy was incorporated for patients up to 40 years of age, and expanded up to 60 years in 2019. The aim of this study is to describe the outcomes of the first 82 consecutive adult Haplo-PTCy transplants between 2016-2021 at the main public center of the Chilean national HSCT program.

## Materials and methods

### Patients

All patients that received an Haplo-PTCy at the Intensive Hematological Unit, Hospital del Salvador, from 2016 to 2021 were included. As mentioned, all cases belonged to the national HSCT program, and were approved by a national adult HSCT committee following a specific Haplo-HSCT protocol established in 2016. Inclusion criteria were age ≥15 and ≤40 from 2016-2018 and ≤60 years old since 2019, Eastern Cooperative Oncology Group (ECOG) performance status <3, no concomitant active cancer, and adequate organ function. For patients with acute leukemias, complete response before transplantation was mandatory. This study was performed according to the Helsinki declaration and was approved by the institutional ethics committee.

### Endpoint and definitions

The main endpoints were event-free survival (EFS), OS, cumulative incidence of relapse (CIR), non-relapse mortality (NRM) at two years and graft-versus-host disease (GvHD)-free, relapse-free survival (GRFS) at one year after transplantation. Secondary endpoints were incidence of Grade II-IV acute graft-versus-host disease (aGvHD) at Day +100 and chronic GvHD (cGvHD) at two years. OS was calculated from the day of infusion until the last visit or death by any cause. Glucksberg criteria were used for aGvHD [11,12]. Chronic GvHD was graded using the National Institutes of Health (NIH) criteria [13]. NRM was defined as death from any cause other than relapse. EFS was calculated from the day of infusion until the day of relapse, graft failure or death. GRFS was defined as one year post-transplant survival without Grade III-IV aGvHD, systemic therapy required for cGvHD, relapse, or death.

The haploidentical donor was defined based on molecular techniques for HLA-A, HLA-B and HLA-DRB1 loci. Disease stage at the time of transplantation was classified by the Disease Risk Index (DRI) [14]. The hematopoietic cell transplantation-specific comorbidity index (HCT-CI) was used to stratify patients according to pre-transplant comorbidities [15].

Myeloablative conditioning (MAC) was defined as a regimen containing either total body irradiation with a dose greater than 6 Gy, a total dose of oral busulfan greater than 8 mg/kg bodyweight, or a total dose of intravenous busulfan >6.4 mg/kg bodyweight [16].

Cytokine release syndrome (CRS) was defined as post-infusion fever up to Day +6, with no clinical focus nor microbiologic agent identified and classified according to Lee (Supplementary Table 1) [17]. Neutrophil engraftment was defined as the first day of an absolute neutrophil count ≥0.5 × 10^9^/L lasting for three or more consecutive days and platelet engraftment as ≥20.0×10^9^/L for five consecutive days without transfusional support. Graft failure (GF) was defined as either lack of initial engraftment of donor cells (primary graft failure) or loss of donor cells after initial engraftment (secondary graft failure) with donor chimerism ≤5 %. Engraftment syndrome (ES) was defined as the presence of fever, weight gain, skin rash, and/or respiratory distress according to Spitzer classification [18]. Poor graft function (PGF), as frequent dependence on blood and/or platelet transfusions and/or growth factor support with donor chimerism >5 % in the absence of relapse, drugs, or infections [19]. Quality of life was evaluated based on Karnofsky performance scale.

### Treatment

Hematopoietic progenitors were obtained from unmanipulated peripheral blood mobilization. The best donor was selected, prioritizing negative specific anti-HLA antibodies (DSA) and crossmatch: age <40 years, male gender, ABO compatibility and lower parity of female donor. The conditioning protocols are shown in Table 1 [20, 21, 22, 23].

**Table 1:** Patient and transplantation characteristics (n = 82).

	n, (%)
Age, median (years) - n (range)	25 (15-51)
Male sex - n (%)	56 (68)
Disease - n (%)	
ALL	47 (57)
AML/MDS	25 (31)
SAA	3 (4)
PNH	2 (2)
HL	2 (2)
CML	2 (2)
BPDCN	1 (1)
DRI - n (%)	
Low	9 (11)
Intermediate	46 (56)
High	25 (30)
Not assessed	2 (2)
HCT-CI - n (%)	
Low	60 (75)
Intermediate	17 (21)
High	4 (5)
Type of conditioning - n (%)	
Flu (120 mg/m^2^) TBI (6×2Gy)	34 (41)
Bu (16 mg/kg/PO) Flu (120 mg/m^2^)	26 (32)
Cy (120 mg/kg) TBI (2×6Gy)	10 (12)
Bu (16 mg/kg/PO) Cy (120 mg/kg)	5 (6)
Flu (150 mg/m^2^) Cy (29 mg/kg) ATG (7.5 mg/m2) TBI (2 Gy)*	4 (5)
Bu (8 mg/kg/oral) Flu (150 mg/m^2^) Cy (29 mg/kg)*	3 (4)
Donor relationship - n (%)	
Sibling	49 (60)
Parent	17 (21)
Other	16 (19)
Donor-Specific Antibodies - n (%)	
Negative	71 (87)
Positive	1 (1)
Not assessed	10 (12)
ABO Incompatibility - n (%)	
No/Minor	69 (84)
Major/bidirectional	13 (16)
Donor/Receptor CMV status - n (%)	
D+/R+	70 (85)
D+/R-	8 (10)
D-/R+	3 (4)
D-/R-	1 (1)

ALL: Acute lymphoblastic Leukemia; AML/MDN: Acute Myeloid Leukemia/myelodysplastic neoplasms; SAA: Severe Aplastic Anemia; CML: Chronic Myeloid leukemia; PNH: Paroxysmal Nocturnal Hemoglobinuria; HL: Hodgkin Lymphoma; BPDCN: Blastic Plasmacytoid Dendritic Cell Neoplasm; PBSC Peripheral Blood Stem Cell; *: Reduced Intensity Conditioning; PO: orally; FLU Fludarabine; TBI Total Body Irradiation; Bu Busulfan; ATG Thymoglobulin; Cy Cyclophosphamide; GVHD Graft versus Host Disease; CNI Calcineurins; MMF Mycophenolate Mofetil; PT-Cy Post Transplant Cyclophosphamide; D donor; R recipient.

Patients were hospitalized in individual isolation units with positive pressure and 4th generation high efficiency particulate air filters and were assisted by a multidisciplinary Intensive Hematology team. According to the institutional protocol, filgrastim 300 mcg/d sc was universally used from Day +5 until absolute neutrophil count (ANC) >1000. Additional filgrastim, erythropoietin and eltrombopag were allowed if sustained or progressive cytopenias due to infections, drug toxicity, PGF or GF were observed.

Acute GvHD prophylaxis was performed with cyclosporine aiming at levels between 250 and 350 ng/mL from Day +5, mycophenolate 1 g every 8 h orally from Day +5 to Day +35 and in vivo T-cell depletion with cyclophosphamide 50 mg/kg bodyweight iv, on Days +3 and +4 [24].

Antibacterial prophylaxis with ciprofloxacin, acyclovir and fluconazole was used. Febrile neutropenia was managed according to the institutional protocol. Acute GvHD was treated according to its severity. Briefly, global Stage I cases were observed or treated with topical or low-dose systemic steroids (equivalent to prednisone 0.5 mg/kg bodyweight/day). Stage II were treated with standard dose steroids (prednisone 1 mg/kg bodyweight/dose). For Stage III-IV aGvHD, high-dose steroids (prednisone 2 mg/kg bodyweight/dose) were used as first line and calcineurin inhibitors were optimized or restarted. If no response was achieved, mycophenolate or methotrexate was used as second-line treatments. Ruxolitinib was not available during the study period.

Discharge was indicated when engraftment, full oral medication and fluid intake >2.5 L/24 h were achieved. Cyclosporine levels were monitored weekly and cytomegalovirus (CMV) by real time polymerase chain reaction (RT-PCR) biweekly until Day +100. CMV reactivation was defined as a viral load exceeding 1000 copies/mL. CMV disease was diagnosed in cases of clinical signs and symptoms. Preemptive treatment with valganciclovir was used as the first choice in cases of CMV reactivation. Ganciclovir was used in CMV disease. Foscarnet was used in case of severe cytopenias or ganciclovir refractoriness.

### Statistical analysis

All epidemiological data and clinical characteristics of the patients were expressed as frequencies with percentages for categorical variables and the mean with range for numeric variables. OS and EFS were estimated using the Kaplan-Meier method. The cumulative incidence of relapse was calculated using relapse as the primary event and death without relapse as a competing event. R software was used for statistical analysis.

## Results

The characteristics of patients, donors and conditioning regimens are shown in Table 1. Eighty-five haploidentical transplants were performed during the period of analysis. Three second Haplo-PTCy were excluded. Mean age was 25 years (Range: 15-51 years), 94 % (77/82) were for neoplastic diseases with the most common diagnosis (57 %) being acute lymphoblastic leukemia. Sixty-one percent of patients underwent transplants less than one year after diagnosis and 47 % proceeded to transplant in the first complete response. Most patients (76 %) had low-risk HCT-CI scores. Conditioning was mostly myeloablative (96 %). The median number of CD34^+^ cells infused was 8.02 × 10^6^/kg bodyweight (Range: 2.42-10.02×10^6^/kg bodyweight). No patient needed a desensitization regimen. All patients received aGvHD prophylaxis with the planned protocol. PHSP were used in all patients, including those with non-malignant diseases, to reduce the risk of graft failure and to avoid the risk of SARS-CoV2 transmission during the pandemic [25].

### Early post-transplantation events

Grade 1-2 CRS was seen in 83 % however no Grade 3-4 CRS was observed. Almost all (98 %) patients had mucositis and 61 % needed parenteral nutrition, with a median duration of 11 days (Range: 2-29 days). All patients had at least one FN episode, 56 % with a gastrointestinal focus. Bacteremia was observed in 23 % of the patients, with *Staphylococcus epidermidis* being the most common agent. Eighteen percent of patients had a probable invasive aspergillosis.

Neutrophil engraftment was achieved in 96 % of patients, (Median: Day +17; Range: 11-25). Platelet engraftment (>20 × 10^9^/L) was achieved in 95 %, (Median: Day +19; Range: 9-84) and platelets >50×10^9^/L (Median: Day +19; Range: 10-175). PGF was observed in 11 (13 %) patients. Eltrombopag was used in 37 %.

Criteria for ES were identified in 21 (26 %) patients; two patients required low-dose vasopressor drugs or non-invasive ventilatory support, both for less than 48 hours. All patients had a good response with low dose steroids. No mortality was observed.

The incidence of Grade II-IV aGvHD and Grade III-IV at 100 days were 29 % and 5 %, respectively. Grade IV was not observed (Table 2). The median onset was on Day +31 (Range: 13-83): most (89 %) had a good response to oral prednisone (0.5-1 mg/kg bodyweight) with only three (4 %) receiving 2 mg/kg bodyweight. Mycophenolate was used as the second line in four patients and methotrexate in one.

**Table 2:** Acute Graft-Versus-Host staging (n = 82).

	n (%)
**Skin aGVHD**	**40 (49)**
I	17 (21)
II	12 (15)
III	11 (13)
IV	0 (0)
**Liver aGVHD**	**2 (2)**
I	0 (0)
II	2 (2)
III	0 (0)
IV	0 (0)
**Upper gastrointestinal aGVHD**	**30 (37)**
I	27 (33)
II	3 (4)
III	0 (0)
IV	0 (0)
**Lower gastrointestinal aGVHD**	**4 (5)**
I	1 (1)
II	2 (2)
III	1 (1)
IV	0 (0)

Data is presented in simple rates at Day +100, and using the Glucksberg criteria (see text).

aGVHD: Acute Graft-versus-Host Disease.

The present cohort showed a two-year incidence of cGvHD of 41 % (31/75) with a median time of presentation on Day +187 (Range: 112-562). Nine (12 %) patients developed moderate-to-severe cGvHD requiring systemic treatment. Overall, complete response was observed in 25 (33.3 %), and partial response in 5 (6.7 %) patients with one patient presenting progressive disease. There were two cGvHD -related deaths, one due to refractory pulmonary cGvHD and one with systemic progression due to poor treatment compliance.

Cytomegalovirus (CMV) reactivation was observed in 63 % (50/82) of patients at a median of Day +37 (Range: 15-77). Of these patients, four developed CMV disease. The median viral load for these reactivations was 1595 copies/mL (Range: 39-87,000). Treatment with valganciclovir was effective in 66 % of cases. Four patients required treatment with ganciclovir, and one patient received foscarnet. Three patients (4 %) developed post-transplant lymphoproliferative disorder, with one requiring chemotherapy.

### Main endpoints

**Fig. 1:**
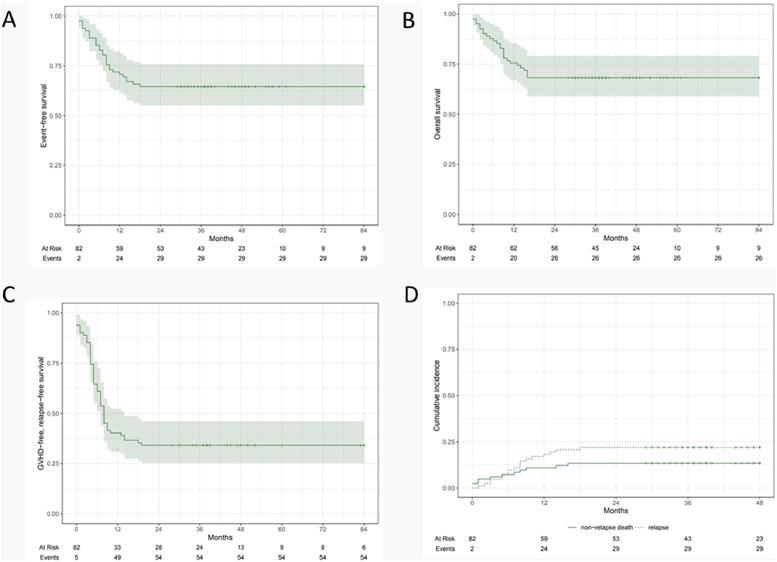
Overall survival (A), event-free survival (B), GVHD-free, relapse-free survival (C) and non-relapse mortality (D).

With a median follow-up of 33 months (Range: 1-84), the estimated three-year EFS and OS of the whole cohort were 64.6 % (95 % confidence interval [95 % CI]: 55-76 %) and 68.3 % (95 % CI: 59-79 %), respectively. Patients with neoplastic disease (n = 77), had a three-year CIR of 23 % (95 % CI: 15-33 %). The two-year NRM cumulative incidence was 13.4 % (95 % CI: 12.2-14.8 %)**.** GRFS at one year was 40.2 % (Figure 1).

### Cause of death

As a whole, 26 patients died, 16 due to relapse at a median Day +250 (Range: 81-510), eight due to transplant related mortality (three to sepsis before engraftment from Klebsiella pneumoniae carbapenemase-producing bacteria when specific antibiotic therapy was not available, two due to secondary GF, one on Day +53 due to aGvHD and two on Days +273 and +276 due to cGvHD). Two patients died due COVID-19 pneumoniae on Days +97 and +228.

## Discussion

This is the first account of Haplo-PTCy in the public health system in Chile. It reports the experience in 82 consecutive patients, showing that it is a feasible and safe procedure to be considered in the absence of an MSD.

These results are encouraging, similar to other adult cohorts using peripheral blood stem cells [7,25, 26, 27, 28, 29, 30]. The two-year OS in those studies range between 57- 68 %, while our cohort shows a 3-year OS of 68 %. Regarding aGvHD, the incidence of Grade II-IV aGvHD was also consistent with the results previously reported by other groups, ranging from 18–42 % globally, and 8–14 % for Grade III-IV, compared with 29 % and 5 % in the preset study, respectively. The two-year cumulative NRM incidence of 0.134 is also similar to that reported in the same studies (16–28 %), as well the two-year CIR of 0.22 (Range: 17–36 %). Finally, our study also shows a good quality of life after Haplo-PTCy with a one-year GRFS of 40 %, compared with 23–43 % in the literature.

When comparing the OS, EFS, CIR and GRFS of the current study to the aforementioned reports, one must consider that this was a carefully selected cohort of young patients. The median age was 25 years (compared to 41-60 years in other studies), and all patients met strict response criteria before transplantation (every patient with acute leukemia was in CR). These variables have been consistently identified as significant good risk factors for OS and EFS after HSCT [15,31,32]. Furthermore, studies of Haplo-PTCy in children with acute lymphoblastic leukemia, with a median age of 10-12 years, from Spain and China, have shown OS of 59–82 % resembling the OS of our young cohort [33,34]. Another factor could be the young age of most of the donors, with a median of 29 years of age (Range: 15-63). Younger donors have also been associated with better outcomes in the Haplo-PTCy setting [35].

This study has several limitations. As mentioned, it is a single-center cohort with a relatively small number of highly selected patients, and it is not possible to compare directly with prior studies. Additionally, we could not compare it with other types of donors, namely MSD (low number of cases) or MUD (not available).

## Conclusions

In conclusion, the experience of this center adds to the evidence that Haplo-PTCy is a safe and effective allogeneic transplant option, when following strict inclusion criteria. The results are comparable to the literature and stand out as the center is a public institution in a LMIC characterized by less investment and facilities in public health.

## Author contribution

BP: Concept/design, Data collection, Data analysis/interpretation, Drafting article, Critical revision of article, Approval of article, Statistics; FB: Data collection, Critical revision of article, Approval of article; JM: Concept/design, Drafting article, Critical revision of article, Approval of article; RB: Data analysis/interpretation, Drafting article, Critical revision of article, Approval of article, Statistics; AA: Concept/design, Critical revision of article, Approval of article; AM Concept/design, Critical revision of article, Approval of article; MEC: Data analysis/interpretation, Drafting article, Critical revision of article, Approval of article; MK: Concept/design, Data analysis/interpretation, Drafting article, Critical revision of article, Approval of article

## Conflicts of interest

The authors declare no conflicts of interest.

## Appendix Supplementary materials

Image, application 1
