# Supplementary material for: Haploidentical hematopoietic stem cell transplantation with post-transplant cyclophosphamide in the public Chilean national health system: A single center study
Source: Hematol Transfus Cell Ther. 2025 Sep 17;47(4):103982. doi: 10.1016/j.htct.2025.103982 (PMC12476062; doi:10.1016/j.htct.2025.103982)
Supplement: Supplementary file 1 [file mmc1.docx]

| **Supplementary Table 1:** Cytokine release syndrome (CRS) classification | |
| --- | --- |
| **Grade** | **Toxicity** |
| **Grade 1** | Symptoms are not life threatening and require symptomatic treatment only, e.g., fever, nausea, fatigue, headache, myalgias, malaise |
| **Grade 2** | Symptoms require and respond to moderate intervention |
|  | Oxygen requirement <40% or |
|  | Hypotension responsive to fluids or low dose of one vasopressor or |
|  | Grade 2 organ toxicity |
| **Grade 3** | Symptoms require and respond to aggressive intervention |
|  | Oxygen requirement ≥40% or  Hypotension requiring high dose or multiple vasopressors or |
|  | Grade 3 organ toxicity or grade 4 transaminitis |
| **Grade 4** | Life-threatening symptom |
|  | Requirement for ventilator support |
|  | Grade 4 organ toxicity (excluding transaminitis) |
| **Grade 5** | Death |

according to Lee et al. [17]

Grades 2-4 refer to Common Terminology Criteria for Adverse Events v4.0 grading.
